# Electrochemotherapy as a First Line Treatment in Recurrent Squamous Cell Carcinoma of the Oral Cavity and Oropharynx PDL-1 Negative and/or with Evident Contraindication to Immunotherapy: A Randomized Multicenter Controlled Trial

**DOI:** 10.3390/cancers13092210

**Published:** 2021-05-04

**Authors:** Francesco Perri, Francesco Longo, Roberta Fusco, Valeria D’Alessio, Corrado Aversa, Ettore Pavone, Monica Pontone, Maria Luisa Marciano, Salvatore Villano, Pierluigi Franco, Giulia Togo, Gianluca Renato De Fazio, Daniele Ordano, Fabio Maglitto, Giovanni Salzano, Maria Grazia Maglione, Agostino Guida, Franco Ionna

**Affiliations:** 1 Medical and Experimental Head and Neck Oncology Unit, Istituto Nazionale Tumori IRCCS Fondazione Pascale-IRCCS di Napoli, Via M. Semmola, 80131 Naples, Italy; f.perri@istitutotumori.na.it (F.P.); m.pontone@istitutotumori.na.it (M.P.); m.marciano@istitutotumori.na.it (M.L.M.); 2 Head and Neck Surgery Unit, Ospedale Casa Sollievo della Sofferenza, S. Giovanni Rotondo, 71013 Foggia, Italy; maxillofacciale@operapadrepio.it; 3 IGEA SpA Medical Division—Oncology, Via Casarea 65, Casalnuovo di Napoli, 80013 Napoli, Italy; v.dalessio@igeamedical.com; 4 Head and Neck Surgery Unit, Istituto Nazionale Tumori IRCCS Fondazione Pascale-IRCCS di Napoli, Naples, Via M. Semmola, 80131 Naples, Italy; c.aversa@istitutotumori.na.it (C.A.); e.pavone@istitutotumori.na.it (E.P.); s.villano@istitutotumori.na.it (S.V.); p.franco@istitutotumori.na.it (P.F.); f.maglitto@istitutotumori.na.it (F.M.); g.salzano@istitutotumori.na.it (G.S.); m.maglione@istitutotumori.na.it (M.G.M.); f.ionna@istitutotumori.na.it (F.I.); 5 School of Specialization in Maxillo-Facial Surgery, University of Naples Federico II, Via Sergio Pansini, 80131 Naples, Italy; g.togo@istitutotumori.na.it (G.T.); g.defazio@istitutotumori.na.it (G.R.D.F.); d.ordano@istitutotumori.na.it (D.O.); 6 U.O.C. Odontostomatologia, AORN A. Cardarelli, 80131 Naples, Italy; a.guida@istitutotumori.na.it

**Keywords:** electrochemotherapy, recurrent cell squamous carcinoma, randomized trial

## Abstract

**Simple Summary:**

A large number of patients with head and neck squamous cell carcinoma (HNSCC) have an advanced-stage disease (stages III to IVB) that do not respond to therapy despite aggressive, site-specific multimodality therapy and most of them will develop disease recurrence. This is the description of a phase IIb randomized multicenter trial that involves the enrolment of 96 patients. The aim of the study is to verify whether electrochemotherapy performed with bleomycin of head and neck squamous cell carcinoma (HNSCC) relapses of the oral cavity and oropharynx is able to lead to an increase in the objective response rate in comparison with the systemic treatment with cetuximab + platinum-based therapy + 5-fluorouracil. The primary objective is to verify the objective response rate of patients in the control arm compared to the treatment arm.

**Abstract:**

Background: A significant proportion of patients with head and neck squamous cell carcinoma (HNSCC) have advanced-stage disease (stages III to IVB) that do not respond to therapy despite aggressive, site-specific multimodality therapy. A great number of them will develop disease recurrence, with up to 60% risk of local failure and up to 30% risk of distant failure. Therapy can be very demanding for the patient especially when important anatomical structures are involved. For these reasons, therapies that preserve organ functionality in combination with effective local tumor control, like electrochemotherapy (ECT), are of great interest. Until few months ago, systemic cetuximab + platinum-based therapy + 5-fluorouracil represented the standard treatment for HNSCC relapses with a median overall survival of 10.1 months and an objective response rate of 36%. Recently the results of KEYNOTE-048 study were published and a new combination of monoclonal antibody named pembrolizumab and chemotherapy emerged as standard first line therapy of recurrent or metastatic tumor that overexpress tissue PDL-1 (Programmed Death 1 ligand). Nevertheless, a variable percentage from 10 to 15% of patients with recurrent/metastatic disease have a tumor that does not overexpress tissue PDL-1, and therefore, according to the results of the KEYNOTE-048 study, does not benefit from replacement of cetuximab with pembrolizumab. These patients will be treated with the “gold standard”: cetuximab, cisplatin/carboplatin and 5-fluorouracil. Aim: To verify whether electrochemotherapy performed with bleomycin of HNSCC relapses of the oral cavity and oropharynx (single relapse on T) is able to lead to an increase in the objective response rate in comparison with the systemic treatment with cetuximab + platinum-based therapy + 5-fluorouracil in patients with PDL-1 negative tumors. Methods: The phase IIb study involves the enrolment of 96 patients who meet the inclusion criteria (48 in the control arm and 48 in the treatment arm). The control arm involves the treatment of HNSCC with systemic treatment (cetuximab + platinum-based therapy + 5-fluorouracil). The treatment arm involves the ECT with bleomycin. The primary objective is to verify the objective response rate of patients in the control arm compared to the treatment arm.

## 1. Introduction

Electroporation (EP) is a technique in which a short and intense electrical field is applied to cells in order to increase the permeability of the cell membrane. As a consequence of the electroporation, molecules that usually cannot enter the cell membrane can reach the cytoplasm through either diffusion or active transport. The combination of cytotoxic drug and EP is called electrochemotherapy (ECT). This study will use the CLINIPORATOR™ (IGEA S.p.A., Carpi, Italy) which induces electroporation by applying electric pulses, thus allowing the transmembrane and intracellular transfer of molecules or compounds for which cell membranes are normally resistant or minimally permeable.

The first electrochemotherapy clinical trials took place at the Institute Gustave Roussy (Villejuif, France) [1,2,3,4]. The Standard Operating Procedures for electrochemotherapy with the Cliniporator™ were established as a result of a European Commission funded project within the 5th framework program [4,5,6,7,8,9]. Subsequent studies have confirmed clinical effectiveness with an acceptable safety profile. Mali et al. included 44 studies and 1894 tumor nodules in their review and meta-analysis and reported that electrochemotherapy had significantly higher effectiveness than bleomycin or cisplatin alone [10]. In their study of electrochemotherapy (bleomycin followed by electroporation) in 85 patients, no serious electrochemotherapy-related adverse events were reported by Campana et al. [8]. Most common adverse events were postoperative pain (92%) and skin reactions (18%) that decreased over time [8].

A significant proportion of patients with head and neck squamous cell carcinoma (HNSCC) have an advanced-stage disease (stages III to IVB) [11] that do not respond to therapy despite aggressive, site-specific multimodality therapy. A great number of them will develop disease recurrence, with up to 60% risk of local failure and up to 30% risk of distant failure [12,13].

Since many patients with relapsed or metastatic disease are unresponsive to therapy, morbidity is high with reduced survival. Palliative systemic therapy with doublet, platinum-based therapy, more effective compared with single agents determine an improvement of response rate but not of survival. A combination of monoclonal antibody (cetuximab or pembrolizumab) and chemotherapy emerged as standard first line therapy of recurrent or metastatic tumors [14]. More in particular, according to the results of the Keynote 048 trial, when pembrolizumab was used alone versus the standard cisplatin-5fluorouracil-cetuximab scheme, patients whose tumor did express high level of tissue PDL-1, experienced a significantly higher OS if compared with standard chemotherapy (14.9 vs. 10.7 months, *p* < 0.0086). On the other hand, patients whose tumor does not express tissue PDL-1, should be yet treated with the combination of cetuximab-platinum and 5-fluorouracil [14], as well as patients with evident contraindications to the use of pembrolizumab.

## 2. Trial Rational

Recurrent extended neoplasms of the head and neck especially if they involve important anatomical structures can be challenging for the surgeon and very demanding for the patient. Surgical treatment of these lesions could be disfiguring and followed by functional impairment. Moreover, chemo-radiotherapy can be associated with anatomical and functional complications, and importantly, radiotherapy has often already been performed up-front, thus limiting the possibility of re-doing it.

For these reasons, therapies like ECT, that preserve organ functionality in combination with effective local tumor control, are of great interest [6,7,8,15,16,17,18,19,20].

ECT has proven to be effective in the treatment of tumors of the skin, subcutaneous and mucosal tissue with several advantages including high success rate in local tumor control (74% complete regression, 11% partial response after one treatment) with limited damage to healthy tissue with good cosmetic and functional results. In fact, surgery and radiotherapy could be associated with not only a scar, but also volume loss, retraction and other deformities, even for small tumors. ECT, by leaving the treated tissue in site for resorption by the body, without stroma and vascular destruction, allows progressive healing with limited compromise of aesthetics or loss of function, with limited side effects and advantageous cost/benefit ratio. In fact, the technology involved and the drug used are not too expansive and the treatment may not require hospitalization since it can be performed under local or general anesthesia. Usually only one session of treatment is required.

The aim of the study is to evaluate efficacy of bleomycin electrotherapy in treatment of recurrent tumors located in the oral cavity and oropharynx in a two arms prospective, randomized, controlled, phase IIb clinical trial.

In this study 96 patients will be enrolled, 48 each arm, which meet the inclusion criteria (patients that need palliative chemotherapy for recurrent HNSCC in the absence of lymph node and systemic metastasis). All patients have primary tumors of the oral cavity, rhino or oropharynx with histological confirmation of local recurrence, with no indication for surgery or irradiation. The patients must have a PDL-1 negative HSNCC or evident contraindications to the use of pembrolizumab.

## 3. Trial Design

Multicenter, two arms, prospective, randomized, controlled, phase IIb clinical trial of intravenous administration of bleomycin combined with electroporation in patients with recurrent squamous cell carcinoma of oral cavity and oropharynx PDL-1 negative or with evident contraindications to the use of pembrolizumab. Patients randomized to the treatment arm that either have their disease progress, locally or systemically, at any point during follow-up will be crossed over to the control arm.

A total of 96 patients (48 patients each arm) will be enrolled.

### 3.1. Objectives

#### 3.1.1. Primary Objective

To verify if the treatment with ECT and bleomycin is superior in terms of objective response to treatment with cetuximab + therapy based on platinum + 5-fluorouracil. The clinical response will be evaluated by RECIST 1.1 criteria on CT and/or MR images at 2 months from baseline.

#### 3.1.2. Secondary Objective

Overall survival;

Evaluation of progression free survival time;

Evaluation of the duration of the response (defined by the interval from the first documentation of a complete or partial response and the onset of disease progression or death);

Evaluation of disease control, defined as complete response, partial response and stable disease between the two groups;

The quantification of the impact of treatment on the patient quality of life, in terms of pain reduction and bleeding.

### 3.2. Inclusion and Exclusion Criteria

Before enrolment investigators will assess the eligibility of the patients. When a patient is eligible for participation, detailed study information will be provided. The patients who decide to participate in the study will sign informed consent witnessed by a member of the research group. Inclusion and exclusion criteria are summarized in Table 1.

### 3.3. Control Group Treatment

In the control group, patients whose tumor does not express tissue PDL-1 will be treated with the combination of cetuximab, cisplatin/carboplatin and 5-fluorouracil. In addition, patients with evident contraindications to the use of pembrolizumab, in the opinion of the investigator, can be enrolled in this Protocol.

Carboplatin (AUC 5 IV day 1) or cisplatin (at a dose of 100 mg/m^2^) and cetuximab (at a dose of 400 mg/m^2^), as a 2 h intravenous infusion (loading dose), then 250 mg/m^2^, as an infusion intravenously 1 h per week per day 1 and 5-FU (at a dose of 1000 mg/m^2^ per day for 4 days) every 3 weeks followed by maintenance of cetuximab with 250 mg/m^2^ every week or 500 mg/m^2^ every 2 weeks.

### 3.4. Experimental Group Treatment

Each patient will be subjected to electrochemotherapy with bleomycin. ECT is a combination treatment including the injection of an anticancer drug (bleomycin) followed by the delivery of electric pulses using appropriate electrodes and electric pulses. Bleomycin will be injected intravenously to ensure that the whole tumor field will be infiltrated by the drug and that the concentration of bleomycin in the interstitial tissue will be sufficient to kill all the dividing tumor cells while completely sparing the normal non-dividing cells.

Bleomycin is active on any type of tumor because electroporation affects all the cell types and, once cells are electro-permeabilized, it can enter all the cells and interact with the cell DNA in the same way whatever the cell type and whatever the genetic expression of the cell.

Bleomycin will be prepared in the pharmacy of each Institution using the standard procedures for this drug. It will be injected in an intravenous bolus over a short time, up to 5 min at a dose of 15,000 IU/m^2^.

Appropriate electrodes will be inserted in and around the lesions 8 min after the end of the bolus injection of bleomycin. The electric pulses are generated by a Cliniporator™ (IGEA SpA, Carpi, Italy) and will be delivered by means of appropriate electrodes. Both the electrodes and pulse generator have CE certification. ECT treatment will be performed according to ESOPE protocol [5,6].

#### ECT Procedure

Cliniporator™ (IGEA S.p.A., Italy) will be used to deliver electric voltage with the following parameters: 8–96 pulses of 140–1000 V, of 100 μs duration, at 5000 Hz repetition frequency. The electrodes will be chosen between linear, hexagonal, finger and expandable deployable endoscopic ones depending on the size and tumor localization. Generally, linear needle electrodes will be used to treat cutaneous, lymph node and subcutaneous lesions smaller than 3 cm while use of the hexagonal will be limited in wider tumors. Finger and expandable deployable endoscopic electrodes will be used to reach less accessible lesions, such as those localized in the oro-pharynx (tonsil and base of tongue). The surgeon will perform multiple procedures of the electrode in the tumor tissue covering the complete area of the lesion to be treated and possibly a margin area of free tissue growths of 3–5mm (overtreatment) around the lesion itself. The procedure can require one or more electrode insertion and pulse applications and must be completed within 40 min from the end of bleomycin injection.

If the procedure will be performed under local anesthesia, the last will be induced between bleomycin injection and the beginning of the electrode’s insertion; while if the procedure will be performed under general anesthesia, bleomycin injection will follow the induction of anesthesia itself.

The patients will be randomized 1:1 to ECT or standard treatment.

**Figure 1:**
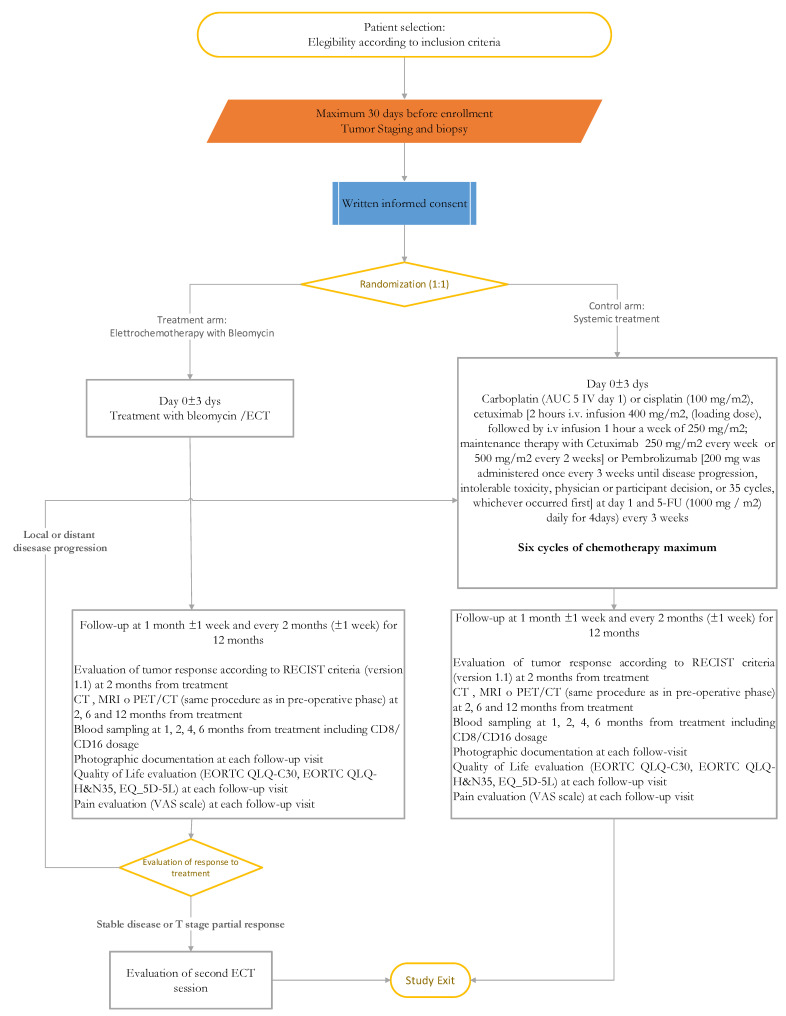
Trial design flowchart.

In Figure 1 the treatment in the two arms of treatment is schematically represented.

### 3.5. Endpoints Evaluation Criteria

#### 3.5.1. Primary Outcome Measures

To verify if the treatment with ECT and bleomycin is superior in terms of objective response to treatment with cetuximab + therapy based on platinum + 5-fluorouracil. Tumor response will be evaluated by means of clinical and radiological examination. Biopsy with histological examination is performed on indication [21].

In case of progression disease (local or systemic): revaluation of the lesion stage will be done and crossover to control arm would be considered. The appearance of one or more new lesions is also considered progression.

In case of stable disease or partial response: revaluation of the lesion stage and the possibility of a second treatment with ECT will be considered.

Objective evaluation of tumor response will be performed in accordance with RECIST criteria (version 1.1) [22].

#### 3.5.2. Analysis of Overall and Progression Free Survival

During the 12-months follow-up period, complete regression duration, appearance of recurrence, causes of death will be recorded. Overall and progression-free survival will be evaluated with Kaplan–Meyer estimation analysis. Overall survival (OS) will be calculated from the date of recruitment to the date of death from any cause or last follow-up. Local Disease Free Survival (LDFS) will be calculated from the date of recruitment to the date of first evidence of local recurrence or last follow-up. Disease recurrence rates and percentages will be compared between two groups. Time of failure of the treatment will be calculated from the date of recruitment to the date of local disease progression. Median and minimum–maximum value will be considered.

QOL, pain and satisfaction questionnaires scores will be computed.

Quality of life will be measured by means of the EORTC QLQ-C30, EORTC QLQ-H&N35, EQ-5D-5L questionnaires at the time of inclusion, 4 and 8 weeks after treatment and at every follow-up visit [23,24,25].

There is no specific toxicity to be expected, with no side effects with the exception of pain and muscular contraction just during the treatment and pain thereafter.

However, specific follow-up will be made during the first 2 post-procedure days in order to register any serious adverse event. The occurrence of an adverse event refers to any clinically significant event that results in death or is life-threatening or that requires the patient’s hospitalization or prolongation of hospitalization.

Power of adverse events not listed in this classification will be evaluated according to the NCI-CTC classification (version 3.0): Mild (grade 1), does not affect the patient’s usual daily activities; Moderate (grade 2), perturbs the patient’s usual daily activities; Severe (grade 3), prevents the patient carrying out his usual daily activities; Very severe (grade 4), necessitates intensive care or is life-threatening; Death (grade 5).

Patients in the treatment group who show disease progression, local or distant, will be assigned to the control arm (Cross-Over). For patients with stable or partial disease a second ECT session may be considered.

The events that eventually occur in the course of the treatment period and/or the follow up, attributable or not to the research, will be considered an adverse event (AE) and will be registered on the applicable case report form. Intercurrent events are any clinical or biological abnormalities not present at the start of the trial. The events will be identified either through patients’ spontaneous statements or following questioning by the researcher and during the examinations.

The National Cancer Institute’s Common Toxicity Criteria for Adverse Events, version 4.0 (CTCAEv4) will be used to estimate the toxicity [26].

### 3.6. Duration of the Study and Follow Up

The study will last 48 months and the patients will be followed up for a minimum of 12 months.

The patients will be visited 1, 2, 4, 6, 8, 10, and 12 months after the treatment. CT/MRI or PET-CT will be performed at 2, 6 and 12 months from treatment. The cut-off point for tumor response evaluation has been fixed at 2 months. The visits scheme is showed in Table 2.

### 3.7. Description of Study Procedures

#### 3.7.1. Visit 1

Patients must sign individual informed consent form before they undergo any study procedures. Patients who fulfil all inclusion criteria and do not meet any exclusion criteria will be enrolled.

At Visit 1, patients will be interviewed and their study eligibility determined according to the inclusion and exclusion criteria and then will be randomized 1:1 to ECT or standard treatment. The investigator will collect and document the patient’s medical history with special emphasis on oncology history, previous chemotherapy, and response to prior therapy. Patients will be asked about concomitant treatments, medications, and events at each patient contact and each visit. Treatment information will include nature of treatment, start date, end date, and reason for treatment. Drug information gathered and documented will include dose, route of administration, frequency, start date, end date, and indication, as applicable. Continuation and discontinuation of treatments and drugs will be verified with the patient at each visit.

The patient will undergo a comprehensive physical examination that includes weight, height, and vital signs. Patients observed with an active infection of any kind will be excluded from study participation.

The ECOG (Eastern Cooperative Oncology Group) Performance Status [27] will be evaluated as inclusion criteria: patients who are grades 0, 1 or 2 are eligible for study participation.

CBC and coagulation profiles will be measured at the enrolment visit (Visit 1). Patients with an absolute neutrophil count below 1000/mL, platelet count below 70,000/mL, and/or INR above 1.5 will be excluded from study participation.

Pain will be assessed at all visits using a 100 mm anchored visual analogy scale with 0 being “No Pain” and 100 mm being “Pain as bad as it could possibly be.”

EORTC QLQ-C30, EORTC QLQ-H&N35, EQ5d questionnaires, will be completed at each follow up visit to measure the patient’s quality of life.

All patients will undergo pre-anesthesia assessment in accordance with the usual and customary anesthesia assessment procedures at the clinical site. Patients found not to be acceptable for either general anesthesia or monitored anesthesia care (conscious sedation) will be excluded from study participation.

#### 3.7.2. Visit 2

After confirmation of continued study eligibility, the day of the treatment the patients will be undergo ECT treatment (treatment group) or directly to cetuximab + platinum + 5-fluorouracil therapy (control group).

Patients randomized to treatment group will undergo ECT treatment in accordance with established clinical and institutional practices.

Pain will be assessed immediately before discharge from the treatment facility. Patients will be encouraged to report symptoms to investigators at any time but will be specifically queried and evaluated for toxicity and adverse events at each visit.

The use of general anesthesia or monitored anesthesia care (conscious sedation with local anesthesia) will be determined by the anesthesiologist based on usual and customary practices for invasive procedures at the respective clinical site.

In the treatment arm, after induction of anesthesia or sedation, the patient will be prepared and draped for the electroporation procedure according to usual and customary procedures for invasive surgical procedures.

Once the lesion has been treated, the usual and customary wound dressing will be applied and the patient will be transferred to post-anesthesia care for further monitoring and pain management. Toxicity and adverse events due to bleomycin will be evaluated after recovery from anesthesia/conscious sedation.

Post-procedure analgesia is prescribed by the investigator in accordance with usual and customary practices at the respective clinical site. Recording of the drugs used for pain control will be done.

The investigator will establish the need for post-procedure hospitalization on the base of the patient’s response to the electrochemotherapy and anesthesia/monitored anesthesia (conscious sedation) care, as well as usual and customary clinical practice at the study site. Routine post procedure hospitalization, therefore, is not considered an adverse event.

#### 3.7.3. Visit 3 (Follow-Up Visit)

Patients will undergo to the same procedures as previously described: clinical evaluation; photographic documentation; EORTC QLQ-C30, EORTC QLQ-H&N35, EQ-5D-5L questionnaires, pain evaluation with VAS score and blood samples as per normal clinical practice and CD8 and CD16 dosages.

#### 3.7.4. Visit 4 (Follow-Up Visit)

The patients will undergo clinical evaluation; photographic documentation; evaluation treatment response according to RECIST criteria (version 1.1); CT, MRI or PET-CT (same imaging as pre-operative evaluation); photographic documentation; EORTC QLQ-C30, EORTC QLQ-H&N35, EQ-5D-5L questionnaires, pain evaluation with VAS score, blood samples as for normal clinical practice and CD8 and CD16 dosages.

Biopsy will be performed only in the case of clinical evidence or radiologic suspect.

#### 3.7.5. Visits 5 and 7 (Follow-Up Visit)

The patients will undergo the same procedures as previously described for visit 3.

#### 3.7.6. Visits 6, 8 and 9 (Follow-Up Visit)

The patients will undergo the same procedures as previously described for visit 4

### 3.8. Description of Sample Size Calculation

Sample size estimation was done by using the superiority hypothesis in two independent parallel sample proportions [28,29,30,31,32,33,34]. Primary endpoint is the evaluation of the objective OR tumor response (CR + PR) based on RECIST criteria between treatment and control arm. According to literature data, treatments with cetuximab determine an OR response rate of about 36%.

We assume that ECT can provide an increase of about 20% of the OR (the EURECA study reports a global H&N response rate of 56% in recurrent, mucosal head and neck tumors).

The test statistic used is the one-sided T-test. The significance level of the test was targeted at 0.15. Group sample sizes of 48 in group one and 48 in group two achieve 81% power to detect a difference between the group proportions of 0.2000. The proportion in group one (the treatment group) is assumed to be 0.36 under the null hypothesis and 0.56 under the alternative hypothesis. The proportion in group two (the control group) is 0.36. The significance level actually achieved by this design is 0.16.

### 3.9. Statistical Analysis

Continuous variables will be presented as mean ± standard deviation if normally distributed; as median and interquartile range (IQR) if not normally distributed. The difference between follow-up values and baseline values will be computed and the null hypothesis (no effect on local disease control and quality of life or pain) will be tested by two-sided Student’s t-test (if differences normally distributed) or by Mann–Whitney test (if differences not normally distributed). ANOVA test (normally distributed variables) or Kruskall–Wallis test (non-normally distributed variables) will be used to test the null hypothesis by adjusting for proper covariates. For categorical variables, the chi square test for multiple comparisons will be used.

The objective response will be reported as a categorical variable and marked as a response rate.

Survival analysis will be performed with the Kaplan–Meier test, and the difference between the curves with the log-rank test.

Quality of life and pain assessment will be evaluated as continuous variables. The difference between the follow-up values and baseline values will be calculated and the null hypothesis (no effect) will be tested with two-tailed Student’s t-test (if the differences are distributed according to a normal one) or with the Mann–Whitney test (if the differences are not distributed according to a normal). The ANOVA test will be used to verify the null hypothesis by adjusting for appropriate covariates (if the differences are distributed according to a normal one) or with the Kruskal–Wallis test.

Differences will be considered significant at *p* < 0.05.

## 4. Discussion

In HNSCC with advanced-stage disease, the drug of choice is platinum combined with fluorouracil (FU) or a taxane; these regimens generally resulted in a 30% response rate, median progression-free survival (PFS) of 3–4 months, and median overall survival (OS) of 6–8 months [20,35,36,37]. Currently, a significantly prolonged median survival from 7.4 to 10.1 months was obtained combining targeted therapy to cytotoxic chemotherapy, pointing out the lack of a lasting effect with most active regimens for patients with recurrent/metastatic HNSCC [38].

Long-term survival is possible in a few numbers of patients with locally recurrent, non-metastatic HNSCC that is candidate to salvage surgery and/or reirradiation [39,40,41,42,43,44]. Some data suggests a longer disease-free survival in patients with locally recurrent disease treated with palliative systemic therapy compared with patients with metastatic disease [42].

Recently the results of KEYNOTE-04, a randomized, multicenter phase 3 study of patients with untreated locally incurable HNSCC tumors showed that a new combination of monoclonal antibody and chemotherapy emerged as standard first line therapy of recurrent or metastatic tumors [14]. After stratification by PD-L1 expression, p16 status, and performance status the patients were randomly allocated (1:1:1) to three arms: pembrolizumab alone, pembrolizumab plus a platinum and 5-fluorouracil (pembrolizumab with chemotherapy), or cetuximab plus a platinum and 5-fluorouracil (cetuximab with chemotherapy). After a median follow-up of approximately 13 months, an improved OS was observed in patients with CPS ≥ 20 (median 14.7 versus 11.0 months, two-year OS 35% versus 19%, hazard ratio (HR) 0.60, 95% CI 0.45–0.82) and in those with CPS ≥1 (median 13.6 versus 10.4 months, two-year OS 31 versus 17%, HR 0.65, 95% CI 0.53–0.80) in the group that received pembrolizumab-containing combination therapy, in comparison with cetuximab-containing combination therapy. No statistically significant improvement of OS in the total study population (median 13.0 versus 10.7 months, two-year OS 29% versus 19%, HR 0.77, 95% CI 0.63–0.93) was present. No relevant differences in progression-free survival in patients with CPS ≥20 (median 5.8 versus 5.2 months, HR 0.73, 95% CI 0.55–0.97) or CPS ≥1 (median 5.0 months each, HR 0.82, 95% CI 0.67–1.00) was observed. Comparable objective response rates (43% versus 38% for CPS ≥20, 36% each for CPS ≥1), was found in patients with any positive CPS (7.1 versus 4.2 months for CPS ≥20, 6.7 versus 4.3 months for CPS ≥1) and a longer duration of response was also observed. The KEYNOTE-048 study showed that combination therapy of pembrolizumab with platinum and 5-fluorouracil significantly is able to improve overall survival in the PD-L1 CPS of 20 or more, CPS of 1 or more, and total populations, and that was associated with a longer duration of response, with a comparable objective response, PFS and safety profile versus cetuximab with chemotherapy. Based on efficacy and safety data, pembrolizumab combined with platinum and 5-fluorouracil can be considered a new standard-of-care treatment for patients with recurrent or metastatic HNSCC. Nevertheless, a percentage of patients ranging from 10 to 15%, has a tumor that does not overexpress tissue PDL-1 (PDL-1 negative), and therefore, according to the results of the aforementioned study, does not benefit from the replacement of cetuximab with the pembrolizumab. In addition, another percent of patients, despite being carriers of PDL-1 positive tumors, has clear and obvious contraindications to the use of immunotherapy, as for example occurs for patients with autoimmune diseases or for patients with chronic viral hepatitis (from virus B or C). In such patients, the standard systemic therapy remains the combination of cetuximab with cisplatin/carboplatin and 5-fluorouracil.

ECT was investigated on 36 patients with recurrent and mucosal head and neck tumors. This prospective multicenter trial [45] showed an objective response of 56% with eight patients in complete response (19%), 16 in partial response (37%), 10 in stable disease (23%). Three patients showed a progressive disease (7%). Three patients (7%) showed a durable complete response at 30, 34, and 84 months post-treatment.

Electrochemotherapy ECT has been shown to be more effective than other therapeutic options in locally advanced SCC treatment. Di Monta et al. [46] in their retrospective, single-center study showed a OR after ECT treatment of stage III SCC of 81% and CR of 22.7%. ECT treatment is generally well accepted by the patients and can be repeated without worsening quality of life of the patients but rather improving their symptoms. ECT resulted in an improvement of symptoms with pain and bleeding reduction also in patients with partial response. The need for medical/paramedical care resulted in a sensible reduction of costs for the hospital in terms of the commitment of medical and nursing personnel [47].

The ECT proved to be an interesting antitumoral therapy in 93 patients with advanced chemo and radio-refractory HN neoplasms [48]. Five percent of patients experienced a complete response, 40% partial responses and 20% of the patients experienced a disease progression after the first ECT procedure. The remaining 34% of patients showed a stable disease. No toxicities related to ECT were seen. ECT is able to reduce frequent symptoms, such as pain and bleeding, especially in patients with moderate symptoms before the treatment, improving quality of life without damage to healthy tissue and with limited side effects. Moreover, ECT reduces hospitalization time and may contribute to an overall reduction in healthcare costs associated with advanced H&N cancers [48].

In a large number of patients with recurrent/metastatic HNSCC, survival outcomes remain poor and a need for new therapeutic options that can prolong the survival and improve quality of life of these patients is urgent.

## 5. Conclusions

This study is the first two arms prospective controlled, randomized, phase IIb clinical trial that will compare efficacy of bleomycin electrotherapy in treatment of recurrent tumors located in the oral cavity and oropharynx with a standard therapy.

## Figures and Tables

**Table 1:** Inclusion and exclusion criteria.

Inclusion Criteria	Exclusion Criteria
Age ≥ 18 years	Age < 18 years
Progressive disease (single recurrence on T and N0) of oral cavity or oropharynx. Absence of other systemic metastases in any site outside the locoregional recurrence	Other symptomatic lesions not under control
Histological diagnosis of squamous cell carcinoma	Lesions not suitable for ECT (bone invasion, large vessels infiltration, etc.)
Measurable lesions suitable for application of electric pulses	Lesions not eligible for systemic treatment with cetuximab + platinum + 5-fluorouracil therapy
Performance status (Karnofsky ≥ 70; WHO ≤ 2)	Acute lung infection
Life expectancy> 3 months	Symptoms of poor lung function
Ability to understand the information given and sign informed consent	Non-correctable severe coagulation disorders
Patients must have offered standard treatments	Previous allergic reactions to bleomycin
	Previous cumulative dose of 250 mg/m^2^ of bleomycin exceeded
	Chronic renal dysfunction (creatinine> 150 µmol/L must be considered a lower administered dose of bleomycin)

**Table 2:** Visits scheme.

Visit		1	2		3	4	5	6	7	8	9
Time		Month -1 (up to 1 month prior the treatment)	Month 0		Month 1 ± 1 week	Month 2 ± 1 week	Month 4 ± 1 week	Month 6 ± 1 week	Month 8 ± 1 week	Month 10 ± 1 week	Month 12 ± 1 week
	Visit Description	Restaging, enrolment, randomization	Day of treatment Control group: cetuximab + platinum + 5- fluorouracil Treatment group: electrochemotherapy	Post treatment evaluation (discharge day ± 1 week)	Follow up visit	Follow up visit Cut off time to evaluate treatment response	Follow up visit	Follow up visit	Follow up visit	Follow up visit	Follow up visit
Type of assessment											
Clinical evaluation			X	X	X	X	X	X	X	X	X
Duration of hospitalization				X							
CT, MRI or PET-CT		X	Estimation of Lesion size and assignment of T stage			X		X			X
Identification of the target lesion		X	X	X							
Photographic documentation		X	X		X	X	X	X	X	X	X
EORTC QLQ-C30, EORTC QLQ-H&N35, EQ-5D-5L questionnaires		X		X	X	X	X	X	X	X	X
Pain evaluation with VAS score		X		X	X	X	X	X	X	X	X
Blood samples as per normal clinical practice		X	X		X	X	X	X	X	X	X
CD8 and CD16 dosage			X		X	X	X	X			
Recording of the drugs for pain control				X		X	X	X	X	X	X
Recording of concomitant treatment					X	X	X	X	X	X	X
ECOG status		X			X	X	X	X	X	X	X
Adverse events/complications					X	X	X	X	X	X	X

## Data Availability

All protocol data are reported in the manuscript.

## References

[1] Belehradek M., Domenge C., Luboinski B., Orlowski S., Belehradek J., Mir L.M. (1993). Electrochemotherapy, a new an-titumor treatment. First clinical phase I-II trial. Cancer.

[2] Mir L. (2006). Bases and rationale of the electrochemotherapy. Eur. J. Cancer Suppl..

[3] Mir L.M., Belehradek M., Domenge C., Orlowski S., Poddevin B., Belehradek J., Schwaab G., Luboinski B., Paoletti C. (1991). [Electrochemotherapy, a new antitumor treatment: First clinical trial]. Comptes Rendus de l’Académie des Sciences Series III Sciences de la Vie.

[4] Marty M., Sersa G., Garbay J.R., Gehl J., Collins C.G., Snoj M., Billard V., Geertsen P.F., Larkin J.O., Miklavcic D. (2006). Electrochemotherapy—An easy, highly effective and safe treatment of cutaneous and subcutaneous metastases: Results of ESOPE (European Standard Operating Procedures of Electrochemotherapy) study. Eur. J. Cancer Suppl..

[5] Mir L.M., Gehl J., Sersa G., Collins C.G., Garbay J.-R., Billard V., Geertsen P.F., Rudolf Z., O’Sullivan G.C., Marty M. (2006). Standard operating procedures of the electrochemotherapy: Instructions for the use of bleomycin or cisplatin administered either systemically or locally and electric pulses delivered by the CliniporatorTM by means of invasive or non-invasive electrodes. Eur. J. Cancer Suppl..

[6] Gehl J., Sersa G., Matthiessen L.W., Muir T., Soden D., Occhini A., Quaglino P., Curatolo P., Campana L.G., Kunte C. (2018). Updated standard operating procedures for electrochemotherapy of cutaneous tumours and skin metastases. Acta Oncol..

[7] Quaglino P., Mortera C., Osella-Abate S., Barberis M., Illengo M., Rissone M., Savoia P., Bernengo M.G. (2008). Electrochemotherapy with intravenous bleomycin in the local treatment of skin melanoma metastases. Ann. Surg. Oncol..

[8] Campana L.G., Valpione S., Mocellin S., Sundararajan R., Granziera E., Sartore L., Chiarion-Sileni V., Rossi C.R. (2012). Electrochemotherapy for disseminated superficial metastases from malignant melanoma. BJS.

[9] Tarantino L., Busto G., Nasto A., Fristachi R., Cacace L., Talamo M., Accardo C., Bortone S., Gallo P., Tarantino P. (2017). Percutaneous electrochemotherapy in the treatment of portal vein tumor thrombosis at hepatic hilum in patients with hepatocellular carcinoma in cirrhosis: A feasibility study. World J. Gastroenterol..

[10] Mali B., Gorjup V., Edhemovic I., Brecelj E., Cemazar M., Sersa G., Strazisar B., Miklavcic D., Jarm T. (2015). Electrochemotherapy of colorectal liver metastases—An observational study of its effects on the electrocardiogram. Biomed. Eng. Online.

[11] Argiris A., Karamouzis M.V., Raben D., Ferris R.L. (2008). Head and neck cancer. Lancet.

[12] Seiwert T.Y., Cohen E.E.W. (2005). State-of-the-art management of locally advanced head and neck cancer. Br. J. Cancer.

[13] Marur S., Forastiere A.A. (2008). Head and Neck Cancer: Changing Epidemiology, Diagnosis, and Treatment. Mayo Clin. Proc..

[14] Burtness B., Harrington K.J., Greil R., Soulières D., Tahara M., de Castro G., Psyrri A., Basté N., Neupane P., Bratland Å. (2019). Pembrolizumab alone or with chemotherapy versus cetuximab with chemotherapy for recurrent or metastatic squamous cell carcinoma of the head and neck (KEYNOTE-048): A randomised, open-label, phase 3 study. Lancet.

[15] Chen C., Smye S., Robinson M., Evans J. (2006). Membrane electroporation theories: A review. Med Biol. Eng. Comput..

[16] Domenge C., Orlowski S., Luboinski B., De Baere T., Schwaab G., Belehradek J., Mir L.M. (1996). Antitumor electrochemo-therapy: New advances in the clinical protocol. Cancer.

[17] Allegretti J.P., Panje W.R. (2001). Electroporation Therapy for Head and Neck Cancer Including Carotid Artery Involvement. Laryngoscope.

[18] Campana L.G., Mocellin S., Basso M., Puccetti O., De Salvo G.L., Chiarion-Sileni V., Vecchiato A., Corti L., Rossi C.R., Nitti D. (2009). Bleomycin-Based Electrochemotherapy: Clinical Outcome from a Single Institution’s Experience with 52 Patients. Ann. Surg. Oncol..

[19] Gargiulo M., Moio M., Monda G., Parascandolo S., Cubicciotti G. (2010). Electrochemotherapy: Actual Considerations and Clinical Experience in Head and Neck Cancers. Ann. Surg..

[20] Burian M., Formanek M., Regele H. (2003). Electroporation therapy in head and neck cancer. Acta Oto-Laryngologica.

[21] Therasse P., Arbuck S.G., Eisenhauer E.A., Wanders J., Kaplan R.S., Rubinstein L., Verweij J., Van Glabbeke M., Van Oosterom A.T., Christian M.C. (2000). New guidelines to evaluate the response to treatment in solid tumors. European Organization for Research and Treatment of Cancer, National Cancer Institute of the United States, National Cancer Institute of Canada. J. Natl. Cancer Inst..

[22] Schwartz L.H., Seymour L., Litière S., Ford R., Gwyther S., Mandrekar S., Shankar L., Bogaerts J., Chen A., Dancey J. (2016). RECIST 1.1—Standardisation and disease-specific adaptations: Perspectives from the RECIST Working Group. Eur. J. Cancer.

[23] Groenvold M., Klee M.C., Sprangers M.A., Aaronson N.K. (1997). Validation of the EORTC QLQ-C30 quality of life questionnaire through combined qualitative and quantitative assessment of patient-observer agreement. J. Clin. Epidemiol..

[24] Sherman A.C., Simonton S., Adams D.C., Vural E., Owens B., Hanna E. (2000). Assessing Quality of Life in Patients With Head and Neck Cancer: Cross-validation of the European Organization for Research and Treatment of Cancer (EORTC) Quality of Life Head and Neck module (QLQ-H&N35). Arch. Otolaryngol. Head Neck Surg..

[25] (1990). EuroQol Group EuroQol—A new facility for the measurement of health-related quality of life. Health Policy.

[26] (2010). National Cancer Institute Common Terminology Criteria for Adverse Events. Version 4.0. https://www.eortc.be/services/doc/ctc/ctcae_4.03_2010-06-14_quickreference_5x7.pdf.

[27] Oken M.M., Creech R.H., Tormey D.C., Horton J., E Davis T., McFadden E.T., Carbone P.P. (1982). Toxicity and response criteria of the Eastern Cooperative Oncology Group. Am. J. Clin. Oncol..

[28] Chow S.C., Shao J., Wang H., Loknjgina Y. (2003). Sample Size Calculations in Clinical Research.

[29] Farrington C.P., Manning G. (1990). Test statistics and sample size formulae for comparative binomial trials with null hypothesis of non-zero risk difference or non-unity relative risk. Stat. Med..

[30] Fleiss J.L., Levin B., Paik M.C. (2003). Statistical Methods for Rates and Proportions.

[31] Gart J.J., Nam J.M. (1988). Approximate Interval Estimation of the Ratio in Binomial Parameters: A Review and Corrections for Skewness. Biometrics.

[32] Lachin J.M. (2011). Biostatistical Methods.

[33] Machin D., Campbell M.J., Tan S.B., Tan S.H. (2008). Sample Size Tables for Clinical Studies.

[34] Miettinen O., Nurminen M. (1985). Comparative analysis of two rates. Stat. Med..

[35] Gibson M.K., Li Y., Murphy B., Hussain M.H., DeConti R.C., Ensley J., Forastiere A.A., Eastern Cooperative Oncology Group (2005). Randomized Phase III Evaluation of Cisplatin Plus Fluorouracil Versus Cisplatin Plus Paclitaxel in Advanced Head and Neck Cancer (E1395): An Intergroup Trial of the Eastern Cooperative Oncology Group. J. Clin. Oncol..

[36] Colevas A.D. (2006). Chemotherapy Options for Patients with Metastatic or Recurrent Squamous Cell Carcinoma of the Head and Neck. J. Clin. Oncol..

[37] Bloom D., Goldfarb P. (2005). The role of intratumour therapy with electroporation and bleomycin in the management of advanced squamous cell carcinoma of the head and neck. Eur. J. Surg. Oncol..

[38] Vermorken J.B., Mesia R., Rivera F., Remenar E., Kawecki A., Rottey S., Erfan J., Zabolotnyy D., Kienzer H.-R., Cupissol D. (2008). Platinum-Based Chemotherapy plus Cetuximab in Head and Neck Cancer. N. Engl. J. Med..

[39] Goodwin W.J. (2000). Salvage Surgery for Patients With Recurrent Squamous Cell Carcinoma of the Upper Aerodigestive Tract: When Do the Ends Justify the Means?. Laryngoscope.

[40] Janot F., De Raucourt D., Benhamou E., Ferron C., Dolivet G., Bensadoun R.-J., Hamoir M., Géry B., Julieron M., Castaing M. (2008). Randomized Trial of Postoperative Reirradiation Combined With Chemotherapy After Salvage Surgery Compared With Salvage Surgery Alone in Head and Neck Carcinoma. J. Clin. Oncol..

[41] De Crevoisier R., Bourhis J., Domenge C., Wibault P., Koscielny S., Lusinchi A., Mamelle G., Janot F., Julieron M., LeRidant A.M. (1998). Full-dose reirradiation for unresectable head and neck carcinoma: Experience at the Gustave-Roussy Institute in a series of 169 patients. J. Clin. Oncol..

[42] Argiris A., Li Y., Forastiere A. (2004). Prognostic factors and long-term survivorship in patients with recurrent or metastatic carcinoma of the head and neck. Cancer.

[43] Plaschke C.C., Gothelf A., Gehl J., Wessel I. (2016). Electrochemotherapy of mucosal head and neck tumors: A systematic review. Acta Oncol..

[44] Bertino G., Sersa G., De Terlizzi F., Occhini A., Plaschke C.C., Groselj A., Langdon C., Grau J.J., McCaul J.A., Heuveling D. (2016). European Research on Electrochemotherapy in Head and Neck Cancer (EURECA) project: Results of the treatment of skin cancer. Eur. J. Cancer.

[45] Plaschke C.C., Bertino G., McCaul J.A., Grau J.J., de Bree R., Sersa G., Occhini A., Groselj A., Langdon C., Heuveling D.A. (2017). European Research on Electrochemotherapy in Head and Neck Cancer (EURECA) project: Results from the treatment of mucosal cancers. Eur. J. Cancer.

[46] Di Monta G., Caracò C., Simeone E., Grimaldi A.M., Marone U., Di Marzo M., Vanella V., Festino L., Palla M., Mori S. (2017). Electrochemotherapy efficacy evaluation for treatment of locally advanced stage III cutaneous squamous cell carcinoma: A 22-cases retrospective analysis. J. Transl. Med..

[47] Pichi B., Pellini R., DE Virgilio A., Spriano G. (2018). Electrochemotherapy: A well-accepted palliative treatment by patients with head and neck tumours. Acta otorhinolaryngologica Italica: Organo ufficiale della Società italiana di otorinolaringologia e chirurgia cervico-facciale. Acta Otorhinolaryngol. Ital..

[48] Longo F., Perri F., Pavone E., Aversa C., Maglione M.G., Guida A., Montano M., Villano S., Daponte A., Caponigro F. (2019). Electrochemotherapy as palliative treatment in patients with advanced head and neck tumours: Outcome analysis in 93 patients treated in a single institution. Oral Oncol..

