# Addendum: Perri et al. Electrochemotherapy as a First Line Treatment in Recurrent Squamous Cell Carcinoma of the Oral Cavity and Oropharynx PDL-1 Negative and/or with Evident Contraindication to Immunotherapy: A Randomized Multicenter Controlled Trial. *Cancers* 2021, *13*, 2210

**DOI:** 10.3390/cancers13143412

**Published:** 2021-07-08

**Authors:** Francesco Perri, Francesco Longo, Roberta Fusco, Valeria D’Alessio, Corrado Aversa, Ettore Pavone, Monica Pontone, Maria Luisa Marciano, Salvatore Villano, Pierluigi Franco, Giulia Togo, Gianluca Renato De Fazio, Daniele Ordano, Fabio Maglitto, Giovanni Salzano, Maria Grazia Maglione, Agostino Guida, Franco Ionna

**Affiliations:** 1 Medical and Experimental Head and Neck Oncology Unit, Istituto Nazionale Tumori IRCCS Fondazione Pascale-IRCCS di Napoli, Via M. Semmola, 80131 Naples, Italy; m.pontone@istitutotumori.na.it (M.P.); m.marciano@istitutotumori.na.it (M.L.M.); 2 Head and Neck Surgery Unit, Ospedale Casa Sollievo della Sofferenza, S. Giovanni Rotondo, 71013 Foggia, Italy; frlongo@hotmail.com; 3 IGEA SpA Medical Division—Oncology, Via Casarea 65, Casalnuovo di Napoli, 80013 Napoli, Italy; r.fusco@igeamedical.com (R.F.); v.dalessio@igeamedical.com (V.D.); 4 Head and Neck Surgery Unit, Istituto Nazionale Tumori IRCCS Fondazione Pascale-IRCCS di Napoli, Naples, Via M. Semmola, 80131 Naples, Italy; c.aversa@istitutotumori.na.it (C.A.); e.pavone@istitutotumori.na.it (E.P.); s.villano@istitutotumori.na.it (S.V.); p.franco@istitutotumori.na.it (P.F.); f.maglitto@istitutotumori.na.it (F.M.); g.salzano@istitutotumori.na.it (G.S.); m.maglione@istitutotumori.na.it (M.G.M.); f.ionna@istitutotumori.na.it (F.I.); 5 School of Specialization in Maxillo-Facial Surgery, University of Naples Federico II, Via Sergio Pansini, 80131 Naples, Italy; g.togo@istitutotumori.na.it (G.T.); g.defazio@istitutotumori.na.it (G.R.D.F.); d.ordano@istitutotumori.na.it (D.O.); 6 U.O.C. Odontostomatologia, AORN A. Cardarelli, 80131 Naples, Italy; a.guida@istitutotumori.na.it

**Comments:** We have some additional considerations about the recent published article by Perri et al. [1] in *Cancers* titled “Electrochemotherapy as a First Line Treatment in Recurrent Squamous Cell Carcinoma of the Oral Cavity and Oropharynx PDL-1 Negative and/or with Evident Contraindication to Immunotherapy: A Randomized Multicenter Controlled Trial”. The primary objective of the clinical trial is to verify the objective response rate of patients included in the two arms: the control arm involves the treatment of head and neck squamous cell carcinoma (HNSCC) with systemic treatment (cetuximab + platinum-based therapy + 5-fluorouracil), while the treatment arm involves the Electrochemotherapy (ECT) with bleomycin. All patients have histologically confirmed local recurrence, and are not eligible to surgery or irradiation. Recently, the results of KEYNOTE-048 study were published and a new combination of a monoclonal antibody named pembrolizumab and chemotherapy emerged as a standard first line therapy for recurrent or metastatic HNSCC that overexpress tissue programmed death 1 ligand (PDL-1). The keynote study was a randomized, phase 3 study of patients with untreated locally incurable HNSCC tumors [2] stratified by PDL-1 expression, p16 status, and performance status and randomly assigned (1:1:1) to pembrolizumab, pembrolizumab plus a platinum and 5-fluorouracil (pembrolizumab with chemotherapy), or cetuximab plus a platinum and 5-fluorouracil (cetuximab with chemotherapy). After a median follow-up of approximately 13 months, an increase in overall survival in patients with CPS (combined positive score) ≥ 20 (median 14.7 versus 11.0 months, two-year OS 35% versus 19%) and in those with CPS ≥ 1 (median 13.6 versus 10.4 months, two-year OS 31 versus 17%) was observed in the pembrolizumab with chemotherapy group, compared with the cetuximab with chemotherapy group. A not statistically significant improvement in OS in the total study population (median 13.0 versus 10.7 months, two-year OS 29% versus 19%) was present. No relevant differences in-progression free survival in patients with CPS ≥ 20 (median 5.8 versus 5.2 months) or CPS ≥ 1 (median 5.0 months each) were observed. Similar objective response rates (43 versus 38 percent for CPS ≥ 20, 36 percent each for CPS ≥ 1), but longer duration of response in patients with any positive CPS (7.1 versus 4.2 months for CPS ≥ 20, 6.7 versus 4.3 months for CPS ≥ 1) were found. Therefore, the study showed that therapy with pembrolizumab combined with chemotherapy is able to improve OS in the PDL-1 CPS of 20 or more, CPS of one or more, and total populations, and that it was associated with a longer duration of response, with a comparable objective response, PFS and safety profile compared to cetuximab with chemotherapy. Based on the observed efficacy and safety, pembrolizumab with platinum and 5-fluorouracil can be considered a new standard-of-care treatment for patients with recurrent or metastatic HNSCC [2].

For these reasons, we requested and obtained the authorization (0051703-28/04/2021-AIFA-AIFA_USC-P), from the Italian Medicines Agency, for a substantial amendment to the clinical trial [1] in order to extend the study also to PDL-1-positive patients. These patients will be treated with a combination of pembrolizumab + systemic treatment (platinum and 5-fluorouracil). Inclusion and exclusion criteria were already described in [2], with the difference that the patients with recurrent HNSCC of the oral cavity and oropharynx should be candidate either to systemic therapy with cetuximab (PDL-1-negative and/or with evident contraindication to immunotherapy) or to immunotherapy with pembrolizumab (PDL-1-positive patients, CPS of 1 or more) [2]. The primary endpoint is to verify whether the treatment with ECT and bleomycin is superior in terms of objective response to treatment with cetuximab + therapy based on platinum + 5 fluorouracil, or alternatively to pembrolizumab + therapy based on platinum + 5 fluorouracil. The clinical response will be evaluated by RECIST 1.1 criteria on Computed Tomography and/or Magnetic Resonance images at 2 months from baseline. 

Moreover, the study design foresees a cross-over to failure from the experimental arm and this would allow, in theory, an interesting treatment sequence, i.e., ECT followed by immunotherapy [3]. Recently, the combination of locoregional/cytoreductive therapeutic methods (such as stereotaxic/hypofractionated radiotherapy and ECT) and immunotherapy is being studied, the rationale of which would be given by a possible “abscopal” immunostimulating effect of cytoreductive treatment [4]. The study by Perri et al. would pave the way for a possible in vivo test related to this therapeutic approach.
